# Continuous-Time ΣΔ ADC with Implicit Variable Gain Amplifier for CMOS Image Sensor

**DOI:** 10.1155/2014/208540

**Published:** 2014-02-18

**Authors:** Fang Tang, Amine Bermak, Amira Abbes, Mohieddine Amor Benammar

**Affiliations:** ^1^College of Communication Engineering, Chongqing University (CQU), Shapingba, Chongging 400044, China; ^2^Hong Kong University of Science and Technology (HKUST), ECE Department, Clear Water Bay, Kowloon, Hong Kong; ^3^School of Computing, University of West Scotland, Paisley, Renfrewshire, UK; ^4^Qatar University, 2713 Doha, Qatar

## Abstract

This paper presents a column-parallel continuous-time sigma delta (CTSD) ADC for mega-pixel resolution CMOS image sensor (CIS). The sigma delta modulator is implemented with a 2nd order resistor/capacitor-based loop filter. The first integrator uses a conventional operational transconductance amplifier (OTA), for the concern of a high power noise rejection. The second integrator is realized with a single-ended inverter-based amplifier, instead of a standard OTA. As a result, the power consumption is reduced, without sacrificing the noise performance. Moreover, the variable gain amplifier in the traditional column-parallel read-out circuit is merged into the front-end of the CTSD modulator. By programming the input resistance, the amplitude range of the input current can be tuned with 8 scales, which is equivalent to a traditional 2-bit preamplification function without consuming extra power and chip area. The test chip prototype is fabricated using 0.18 **μ**m CMOS process and the measurement result shows an ADC power consumption lower than 63.5 **μ**W under 1.4 V power supply and 50 MHz clock frequency.

## 1. Introduction

State-of-the-art CMOS image sensors with mega-pixel resolution prefer using column-parallel quantization scheme in order to reduce power. With moderate conversion speed, hundreds of ADCs operating in parallel can achieve a large data rate while the energy efficiency for each ADC is higher than a single high speed ADC. Several column-parallel architectures were proposed in prior arts such as using single slope ADC [1], cyclic ADC [2], and successive approximation register ADC [3]. Column-parallel sigma delta ADC is supposed to be a good choice. However, due to the high oversampling ratio (>100), the power efficiency of the conventional sigma delta ADC does not have an obvious advantage [4]. The first implemented mega-pixel column-parallel discrete-time sigma delta ADC (DTSD) based CMOS imager was presented in [5]. Low power was achieved by replacing the differential amplifier by single-ended inverter amplifier. However, the architecture shown in [5] suffers from poor power noise rejection and large switching noise injection. The front-end of the ADC in [5] is a switched capacitor circuit, which also requires a high driving ability for the source follower.

In this paper, we report a CIS-purpose column-parallel ADC. The proposed quantization circuit consists of a 2nd order single-ended continuous-time ΣΔ modulator and 2nd order digital integrator based decimation filter. The loop filter is implemented by two resistor-capacitor integrators. The first integrator adopts the conventional differential amplifier which has a high power supply rejection ratio. The second integrator is realized by a single-ended autozeroed inverter amplifier to reduce the power. Additionally, the proposed continuous-time ΣΔ ADC contains an implicit preamplification function without consuming extra power and chip area. By programming the input resistance, the input current amplitude has 8 tunable scales. As a result, the ADC dynamic range can be extended.

This paper is organized as follows.  Section 2 introduces the related work.  Section 3 describes the proposed circuit architecture and implementation.  Section 4 demonstrates the measurement results.  Section 5 delivers the conclusion.

## 2. Imager Architecture and Implementation

### 2.1. Prior Art

The previously reported column-parallel discrete time ΣΔ ADC for CMOS image sensor application achieved low power consumption by replacing the conventional OTA with single-ended inverter amplifier. The implicit class AB feature of the inverter reduces the averaged current while keeping a fast settling speed. There are two major drawbacks of such an architecture. The first one is the low power supply rejection. The inverter amplifier is much more sensitive to the supply noise compared to conventional differential amplifier. Without correlated double sampling (CDS), the power supply rejection ratio (PSRR) in frequency domain is typically only 3 dB. Even with the CDS technique, the PSRR in frequency domain cannot be improved beyond 20 dB (or overall 40 dB after noise power integration) [5].

**Figure 1:**
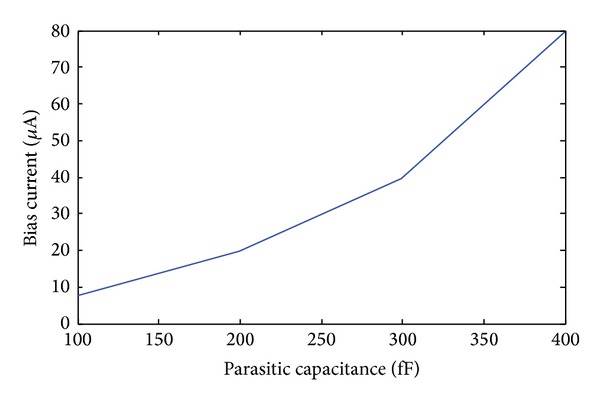
Minimum bias current as a function of the parasitic capacitance.

The second drawback of the architecture in [5] is that the front-end of the discrete time modulator is a switched capacitor circuit. The sampling capacitance must be larger than 45 fF to maintain the KT/C noise better than 11-12 bits [5, 6]. Since the sampling frequency is 50 MHz, the settling time of the source follower should be lower than 20 ns. For a high pixel resolution imager, the parasitic capacitance in the source follower is hundreds of fF; as a result, the high settling speed requires a large bias current. A simulation of the minimum bias current as a function of the parasitic capacitance is shown in Figure 1, where the sampling speed is set to 50 MHz and the sampling capacitance is 45 fF.

In order to overcome the above two drawbacks without decreasing the energy efficiency, we propose a column-parallel continuous-time ΣΔ ADC architecture. On one hand, the supply noise rejection is achieved by using a conventional OTA in the first integrator. On the other hand, the settling issue is greatly relaxed for the proposed scheme since the front-end is a resistance input instead of a switched capacitor input. Further power saving is achieved by merging the preamplifier into the front-end circuit.

### 2.2. Fundament of CTSD ADC

**Figure 2:**
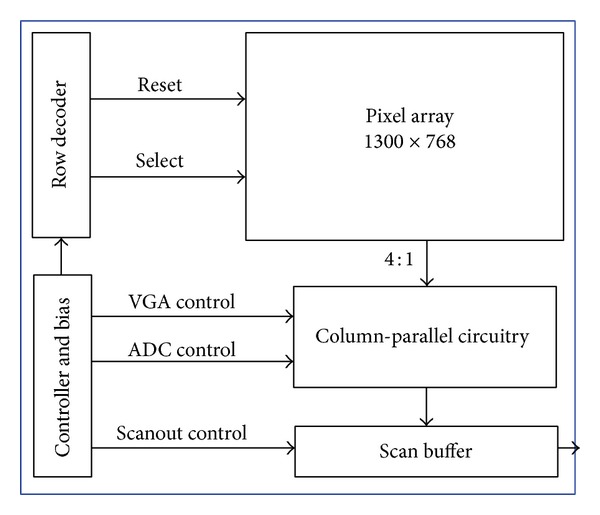
Proposed image sensor architecture.

**Figure 3:**
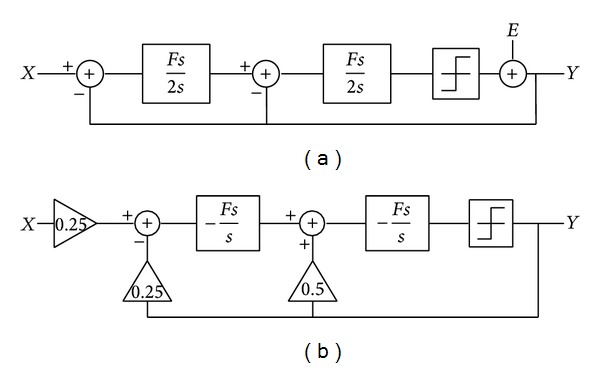
ADC system diagram before (a) and after (b) parameter scaling.

Figure 2 shows the proposed image sensor architecture. It consists of a 1300 × 768 APS pixel array, the row decoders, the controller, the column-parallel ADC, and the scan buffer.  Figure 3 shows the MATLAB system diagram of the ADC before and after parameter scaling. The linearized model of such an ADC could be expressed by (1) in *S* domain, where *H*(*s*) is the transfer function of the internal loop filter. Consider

(1) Y(s)=STF(s)X(s)+NTF(s)E(s),where:{STF(s)=H(s)1+H(s),NTF(s)=11+H(s).

*X*(*s*) is the input signal while *E*(*s*) is the quantization noise induced by the last stage 1-bit quantizer. STF presents the gain factor of the input signal which approximately has an in-band unit gain function. NTF indicates how the quantization noise is attenuated by the noise shaping capability. With RC-based continuous-time integrators, STF and NTF can be derived as (2). Ideally, this 2nd order CTSD ADC can provide 13.38 effect number of bit (ENOB) under 100 oversampling ratios and 50 MHz sampling frequency. Consider

(2) $$\begin{array}{c} \text{STF}\left(s\right)=\frac{H_{\mathrm{int}}{\left(s\right)}^{2}}{H_{\mathrm{int}}{\left(s\right)}^{2}+2H_{\mathrm{int}}\left(s\right)+4},\\ \text{NTF}\left(s\right)=\frac{4}{H_{\mathrm{int}}{\left(s\right)}^{2}+2H_{\mathrm{int}}\left(s\right)+4},\\ H_{\mathrm{int}}\left(s\right)=\frac{Fs}{s}. \end{array}$$

## 3. Proposed System Design

### 3.1. ADC Architecture

**Figure 4:**
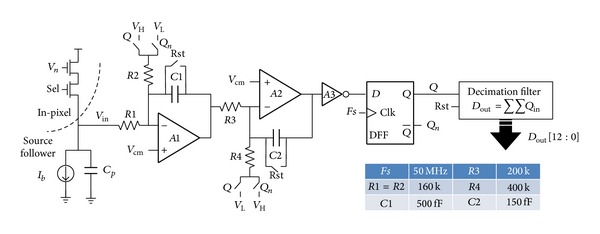
Circuit implementation of the proposed ADC.

The system diagram of the 2nd order continuous-time ΣΔ ADC is shown in Figure 4. The pixel voltage signal *V*_*n*_ is buffered by the source follower with a bias current *I*_*b*_ (20 *μ*A). Then, the voltage *V*_in_ will be converted into a current via the input resistor *R*1 and the feedback signals *Q*/*Q*_*n*_ are also converted into current through *R*2. These two current signals are added in analog domain and then are integrated into the capacitor *C*1. Similarly, *R*3, *R*4, *C*2, and *A*2 form the second integrator. *A*3 is a digital inverter. On one hand, *A*3 isolates the integrator and the latch comparator to reduce the kickback noise; on the other hand, *A*3 provides more than 100 voltage gains which can reduce the quantization noise of the latch comparator.

### 3.2. Implicit Front-End Variable Gain Amplification

**Figure 5:**
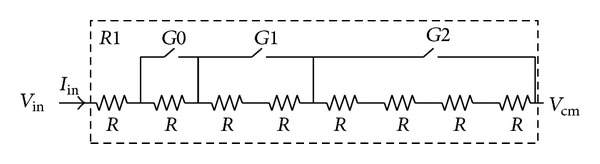
Implementation of the input resistor *R*1.

The front-end input resistor is implemented with a high resistance p+ polyresistor, which is shown in Figure 5. By programming the binary control word *G*, the resistance of *R*1 can be tuned from ×1 *R* to ×8 *R*, where *R* equals 20 kohm. The tunable input resistor is functionally equivalent to a conventional preamplifier without consuming extra power and chip area. When the input voltage is large, the resistance of *R*1 can be programmed to a large value in order to prevent the input current signal saturated. If the input voltage is small, *R*1 is accordingly tuned to be small. As a result, the current *I*_in_ is increased to obtain a well signal-to-noise ratio (SNR). This tunable input resistor can increase the SNR when the input signal is relatively small; thus, the dynamic range of the proposed ADC is extended.

**Figure 6:**
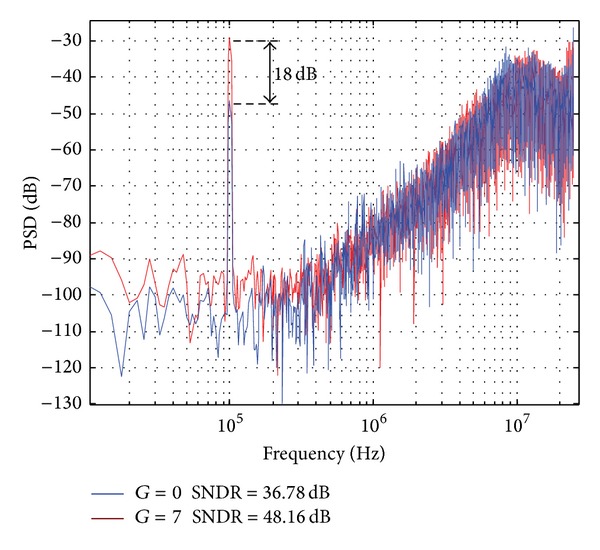
Simulated signal power enhancement.

Assuming the input voltage *V*_in_ has a 10 mV amplitude with 100 kHz frequency, the input current *I*_in_ amplitude can be tuned from 62.5 nA to 500 nA when the binary word *G* is programmed from 0 to 7. Thus, the amplitude of *I*_in_ at the 100 kHz frequency tone should ideally be improved by 18.06 dB, as expressed by (3).  Figure 6 shows the simulated signal power after FFT, indicating a substantial agreement with (3). Consider

(3) $$\begin{array}{c} \text{SN}{\text{R}}_{\text{improved}}=10\log{\left(\frac{V_{\text{in}}}{R}\right)}^{2}-10\log{\left(\frac{V_{\text{in}}}{8R}\right)}^{2}. \end{array}$$

For a *R*1 = 8*R*, the input current noise power density *I*_*n*_^2^ could be expressed by (4), where *C*_*p*_ is the equivalent parasitic capacitance at the input negative node of amplifier *A*1. Ideally, *C*_*p*_ = 0 and 2*πf* · 8*RC*_*p*_ term could be ignored because the input node can seem as a virtual ground. Since *R*1 = *R*2 as shown in Figure 4, the feedback current signal *I*_fb_ has a similar noise power density that is equal to *I*_in__*n*_^2^(*f*)|_*R*1=8*R*_. If *R*1 is reduced from ×1 *R* to ×8 *R* by set *G*, ideally the integrated input current noise power $\bar{\text{Nois}{\text{e}}^{2}}$ should be increased by 6.53 dB, as expressed by (5). As a result, a bandwidth independent 11.53 dB SNR improvement of the current signal can be achieved. Consider

(4) $$\begin{array}{c} {{I_{\text{in}}}_{n}^{2}\left(f\right)|}_{R1=8R}=\frac{4k_{B}T}{8R{\left(1+2\pi f\cdot 8RC_{p}\right)}^{2}}, \end{array}$$

(5) Noiseinc2¯=10 lg(∫0BWIinn2|1R+∫0BWIfbn2) −10 lg(∫0BWIinn2|8R+∫0BWIfbn2)=10 lg(9∫0BWIinn2|8R) −10 lg(2∫0BWIinn2|8R)=6.53 dB.

Since the current signal flowing into the integration capacitor gains an SNR enhancement by 11.53 dB as *G* is set from 0 to 7, the proposed programmable input resistor performs as an analog front-end variable gain amplifier, without consuming extra power and chip area. The extended dynamic range equals the SNR improvement; thus, the proposed CTSD ADC pushes the imager sensing range into the smaller signal region without expensive power consumption and chip area cost compared against conventional CMOS image sensor architectures. Simulation result shows 11.5 dB dynamic range extension, presenting an equivalent 2-bit preamplification function. It should be emphasized that 6.53 dB noise increase as shown in (5) is based on the assumption of ignorable amplifier noise. In practice, the noise increase could be smaller than 6.53 dB when the integrator noise is also taken into account. As a result, the measured dynamic range extension is probably greater than 11.53 dB.

### 3.3. VLSI Implementation

In order to reduce the effect of the supply noise, the first amplifier *A*1 is implemented using a conventional differential amplifier with 50 dB open-loop gain. In the second stage, since *A*2 is less sensitive to the supply noise, the PSRR for *A*2 is less important. However, *R*1, *R*2, and *R*3 have similar resistance, and the current drained through the amplifiers *A*1 and *A*2 is comparable. Thus, it is worth to replace *A*2 with an alternative low power amplifier scheme.

**Figure 7:**
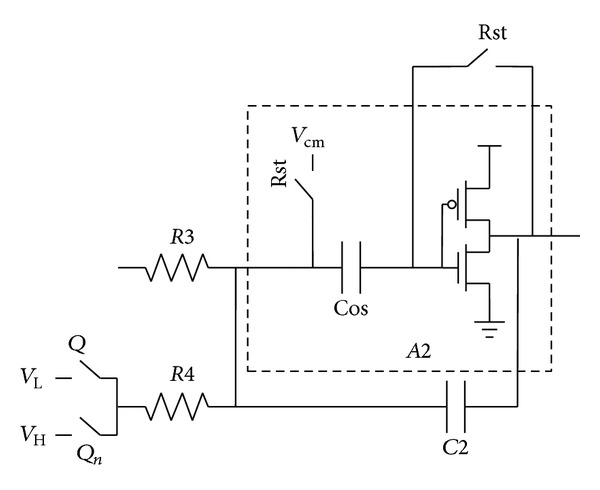
Circuit implementation of *A*2.

**Figure 8:**
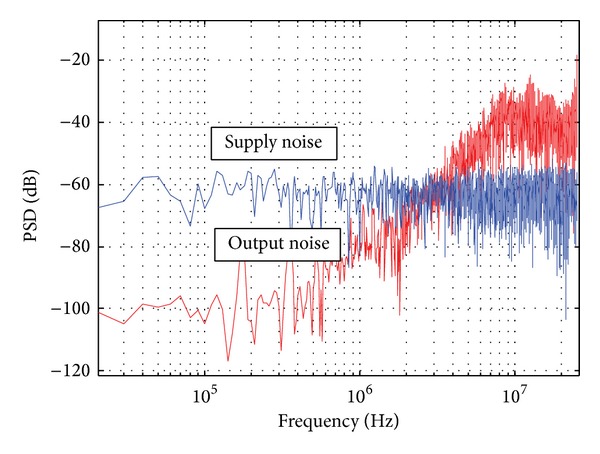
Simulated power supply noise rejection.

In this work, *A*2 can be realized using an inverter amplifier to reduce the power consumption, as shown in Figure 7. During the reset phase, the voltage Vos across the internal capacitor Cos is charged to *V*_cm_ − *V*_*off*⁡_, where *V*_*off*⁡_ is the inverter offset voltage. The autozero technique eliminates the offset variation of the inverter amplifier. By such a class AB inverter-based amplifier, with the same bandwidth, the power consumption is reduced by half. The simulated supply noise rejection of the whole modulator is indicated in Figure 8. A −60 dB power spectrum density noise is injected in the 1.4 V power supply and the simulation result shows about a 40 dB in-band noise attenuation. With digital correlated double sampling technique (DCDS), the PSRR can be further improved by more than 20 dB [5]. The chip layout of the proposed CTSD modulator only occupies 3000 *μ*m^2^ area using 0.18 *μ*m process.

The mathematic function of the decimation filter is a 2nd order digital integrator, as expressed by (6). Consider

(6) $$\begin{array}{c} D_{\text{out}}=\sum^{\text{OSR}}\;\sum^{\text{OSR}}D_{\text{in}}. \end{array}$$

The input of the integrator is a single bit digital signal which is synchronized with the 50 MHz master clock. After 100 cycles, a 13-bit digital value is obtained in the integrator output. The decimation filter is synthesized using digital standard cell and it occupies 10.08 *μ*m × 350 *μ*m area. Totally, 18 D flip-flops and other logic blocks are required for the decimation filter. The scan buffer is implemented by a shift register chain. 13 D flip-flops are used for the scan buffer, consuming extra 10.08 *μ*m × 150 *μ*m area.

**Figure 9:**
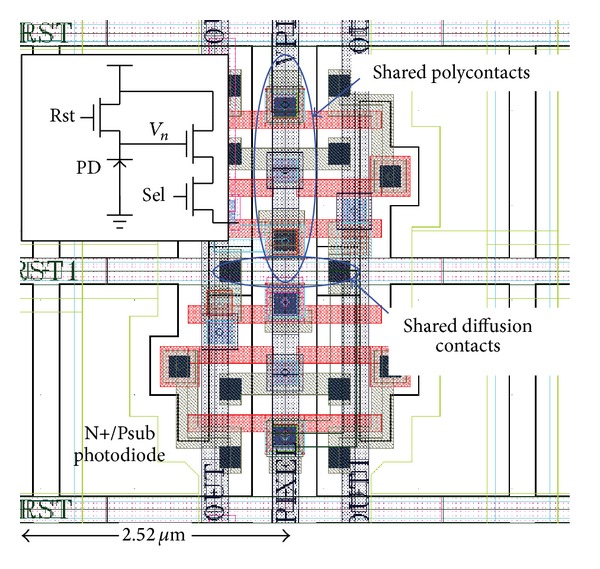
The pixel layout.

The pixel layout is shown in Figure 9. The polycontacts of the Rst/Sel transistors in the horizontal adjacent two pixels are shared, while the diffusion contacts in the vertical adjacent two pixels are also shared. As a result, the photodiode fill factor is increased to 45% with a 2.52 *μ*m pixel pitch.

**Figure 10:**
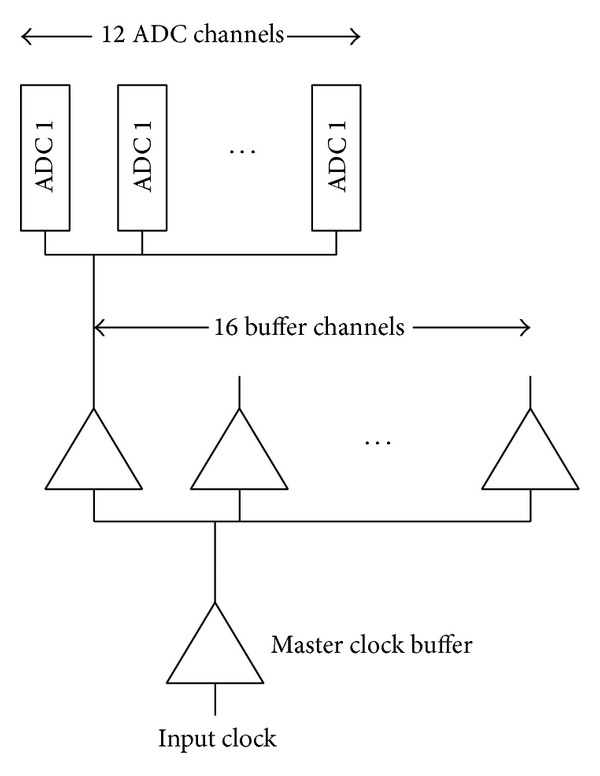
Clock distribution.

The input clock distribution architecture is shown in Figure 10. The whole ADC array is divided into 16 banks and each bank consists of 12 ADC cells. The master clock frequency is 50 MHz. With 100~125 OSR, a several hundreds of ps clock jitter could not obviously affect the ADC ENOB. Since each ADC bank has a 0.5 ns clock propagation delay, each ADC bank can operate with a slight time interval; thus, the superposed clock interference and the digital switching noise are reduced by a factor of 16.

## 4. Experimental Results

**Figure 11:**
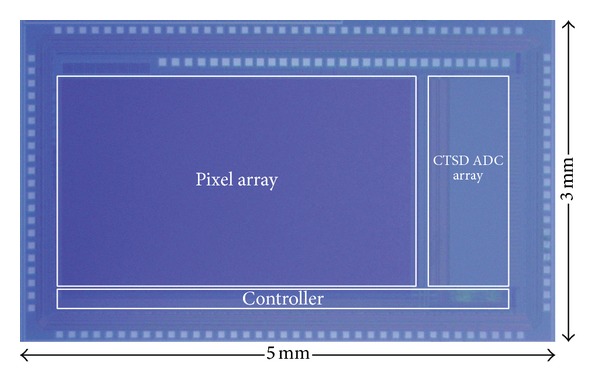
Chip microphotograph.

Figure 11 shows the chip microphotograph. The prototype is implemented using 0.18 *μ*m CMOS process and occupies 5 mm × 3 mm chip area.

**Figure 12:**
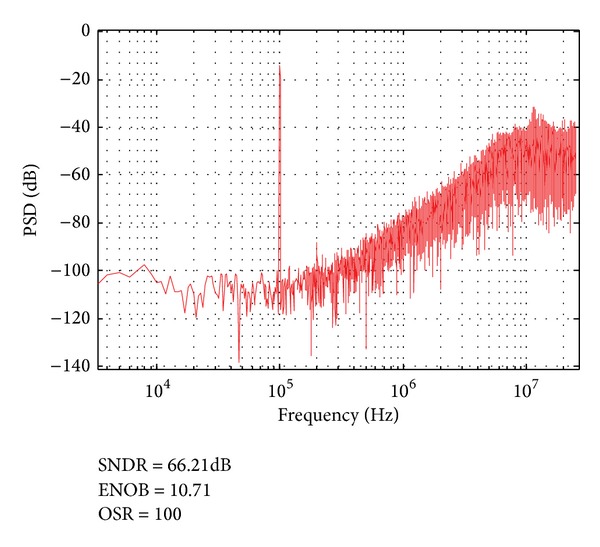
Measured power spectrum density of the proposed CTSD modulator output bit stream (50,000 points FFT).

The measured power spectrum density of the proposed CTSD modulator is shown in Figure 12. The test input signal is a 100 KHz sinusoid voltage with 400 mV amplitude and the master clock frequency is 50 MHz. With 100 oversampling ratios and the front-end resistance control word *G* = 0, 66.21 dB SNDR is achieved, referring to 10.71-bit ENOB. When OSR is set to 128, the sigma delta modulator can provide 11.2-bit ENOB. The realized ENOB is about 3.1 bits lower than the ideal ENOB (13.8 bits) because the resistance of *R*1–*R*4 must be sized with a relatively large value to reduce the power consumption. The averaged power consumption of the proposed modulator is 58.5 *μ*W under a 1.4 V supply voltage. If the proposed system is improved with 100 MHz master clock in the future, smaller resistors can be adopted to achieve smaller thermal noise and higher SNDR.

**Figure 13:**
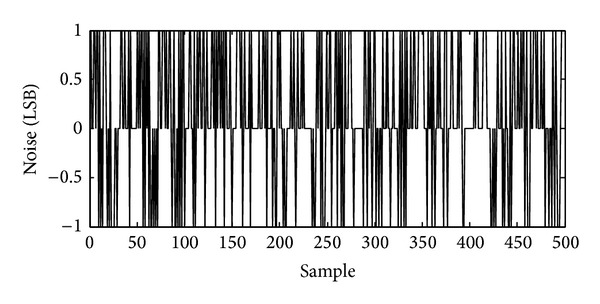
Measured ADC output noise with maximum input signal (500 sample points), indicating 0.7-LSB_RMS_ noise and 72 dB peak SNR.

**Figure 14:**
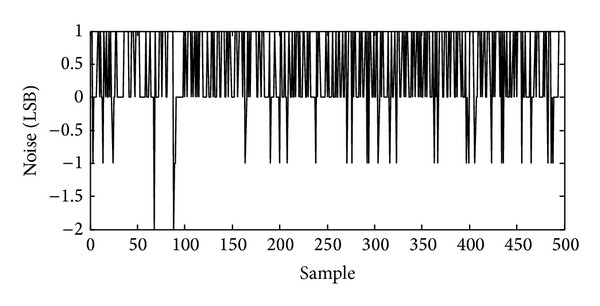
Measured ADC output noise with minimum input signal (500 sample points), indicating 0.6-LSB_RMS_ noise floor and 74 dB dynamic range.

After digitally processed by the decimation filter, the final output code illustrates ±1 LSB peak-to-peak random noise with maximum input signal, as shown in Figure 13. By 500 samples estimation, the root-mean-square (RMS) noise is about 0.7 LSB_RMS_ and 72 dB peak SNR is obtained. With minimum input signal, the noise floor of the ADC output code is about 0.6 LSB_RMS_ as shown in Figure 14. As a result, about 74 dB dynamic range is realized. The column FPN of the total of 192 ADC channels is only 17 LSB after DCDS. The total power consumption of the ADC including both the modulator and decimation filter is about 63.5 *μ*W.

**Figure 15:**
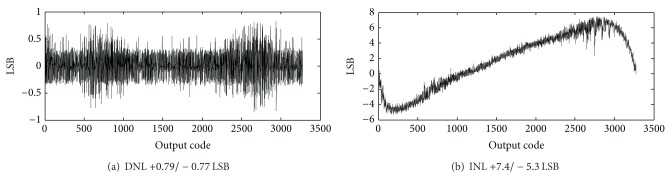
Measured ADC DNL (a) and INL (b).

The differential nonlinearity (DNL) of the proposed CTSD ADC is +0.79/−0.77 LSB, as shown in Figure 15(a). The integrated nonlinearity (INL) of the ADC is +7.4/−5.3 LSB as shown in Figure 15(b), indicating a 0.2% nonlinear error. Since the pixel nonlinear error is normally within 0.5% [5], the INL of the proposed ADC can be ignored.

**Figure 16:**
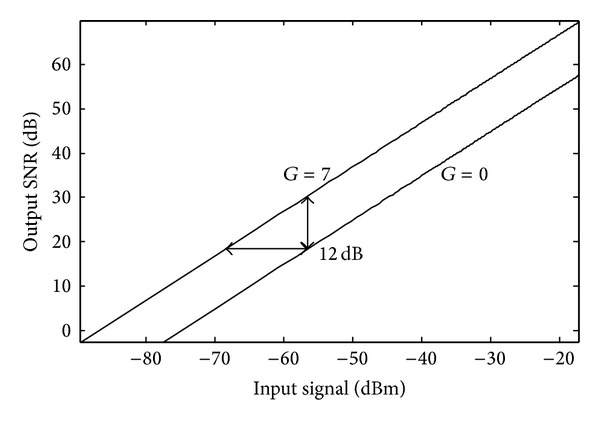
Measured 12 dB ADC dynamic range extension.

To measure the implicit variable gain amplification performance, a set of input signal with a voltage range from 50 mV to 1 mV is provided for the ADC. The achieved SNR as a function of the input amplitude with different values of *G* is shown in Figure 16. By programming *G* from 0 to 7, the output code SNR is enhanced and the dynamic range is extended by about 12 dB, which is consistent with the theoretical derivation in (5). The dynamic range extension of the ADC is equivalent to a 2-bit conventional front-end variable gain amplifier without consuming extra power and chip area.

The proposed CIS-purpose incremental sigma delta ADC can target on state-of-the-art performance especially in two aspects. As expressed in (7), FOM1 is defined as the energy efficiency from the perspective of power consumption, dynamic range, and bandwidth. FOM2 evaluates the chip area utility shown in (8), when taking the chip area, dynamic range, and bandwidth into account. The performance of the proposed ADC is summarized and compared with prior arts as shown in Table 1

(7) $$\begin{array}{c} \text{FOM1}=\frac{\text{Power}}{2\cdot \text{BW}\cdot \text{DR}}, \end{array}$$

(8) $$\begin{array}{c} \text{FOM2}=\frac{\text{Area}}{2\cdot \text{BW}\cdot \text{DR}}. \end{array}$$

## 5. Conclusion

In this paper, a column-parallel 2nd order continuous-time sigma delta ADC for CMOS image sensor is proposed and implemented using 0.18 *μ*m CMOS process. Without consuming extra power and chip area, the ADC dynamic range can be extended from 74 dB to 86 dB by programming the control word *G* from 0 to 7; thus, an equivalent 2-bit front-end variable gain amplifier is realized. The proposed ADC consumes 63.5 *μ*W under 1.4 V supply voltage and 50 MHz clock frequency. 66.21-bit ENOB is achieved for 250 KHz bandwidth with 100 oversampling ratios. For 11-bit quantization resolution, the ADC differential nonlinearity is +0.79/−0.77 LSB.

## Figures and Tables

**Table 1:** CIS-purpose ΣΔ ADC figure-of-merit comparison.

Specification	This work CTSD	JSSC 09 DTSD [7]	JSSC 09 DTSD [7]	JSSC 11 DTSD [5]
Power com. *μ*W	58.5 (ANA) + 5 (DIG)	36 *μ*W (ANA)	5.6 *μ*W (ANA)	40 (ANA) + 3.5 (DIG)
Core area *μ*m^2^	3 k (ANA) + 3.5 k (DIG)	715 k (ANA) + N/A (DIG)	3 k (ANA) + N/A (DIG)	1.35 k (ANA) + 1.35 k (DIG)
Order	2	3	2	2
Peak SNDR	66.21 dB	81 dB	63 dB	—
Peak SNR	72 dB	84 dB	72 dB	66 dB
ENOB	10.71 bits	13.16 bits	10.17 bits	—
Dynamic range	74 dB at *G* = 0	85 dB	76 dB	75 dB
	86 dB at *G* = 7			
Bandwidth	250 KHz	20 KHz	8 KHz	220 KHz
Process	0.18 *μ*m		0.35 *μ*m	0.13 *μ*m
FOM1 fJ/step	25 at *G* = 0	51	55	17
	6.4 at *G* = 7			
FOM2 *μ*m^2^/Hz·step	2.6 at *G* = 0	1000	30	1.1
	0.7 at *G* = 7			
